# Bilateral Asymmetric Rhegmatogenous Retinal Detachment in a Patient with Stickler Syndrome

**DOI:** 10.4274/tjo.60430

**Published:** 2018-04-25

**Authors:** Caner Öztürk, Almila Sarıgül Sezenöz, Gürsel Yılmaz, İmren Akkoyun

**Affiliations:** 1 Başkent University Faculty of Medicine, Department of Ophthalmology, Ankara, Turkey; 2 Çankırı State Hospital, Ophthalmology Clinic, Ankara, Turkey

**Keywords:** Stickler syndrome, rhegmatogenous retinal detachment, scleral buckling, vitrectomy

## Abstract

Here we present the long-term anatomical and visual outcomes of bilateral asymmetric rhegmatogenous retinal detachment repair in a patient with Stickler syndrome. A 17-year-old girl presented with decreased visual acuity in both eyes for more than one year. Her best-corrected visual acuity (BCVA) was 0.1 in the right eye and 0.05 in the left eye. Slit-lamp anterior segment examination revealed subcapsular cataract in both eyes. Fundus examination showed bilateral rhegmatogenous retinal detachment, chronic retinal detachment accompanied by multiple retinal holes, tears and membranous fibrillary vitreous in the peripheral retina. Grade C2 proliferative vitreoretinopathy was observed in the left eye. Scleral buckling surgery was performed initially for both eyes. After the primary surgical procedure, retinal reattachment was achieved in the right eye and the left eye underwent phacoemulsification, intraocular lens implantation, pars plana vitrectomy (PPV), and silicone oil injection. After these surgical procedures retinal reattachment was achieved in the left eye. Silicone oil removal was performed six months after PPV surgery. After surgical treatment, BCVA was 0.6 in the right eye at the end of the 3.5-year follow-up period. After silicone oil removal, BCVA reached 0.2 in the left eye after 36 months of follow-up and retinal reattachment was achieved in both eyes. Scleral buckling surgery and PPV are effective and confidential methods for the treatment of chronic retinal detachment cases in Stickler syndrome.

## Introduction

Stickler syndrome is an autosomal-dominant hereditary clinical condition caused by structural abnormalities in collagen types 2, 9, and 11. It can manifest with widely varying clinical signs affecting the ocular, orofacial, musculoskeletal, and/or auditory systems.^1,2,3,4^ Ophthalmologic complications of the syndrome are progressive and can lead to blindness.^5^ In this report, we present a case of bilateral asymmetric rhegmatogenous retinal detachment associated with Stickler syndrome and the outcomes of treatment.

## Case Report

**Figure 1:**
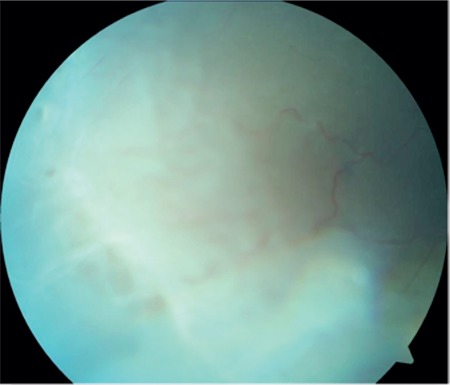
Preoperative appearance of the right retina in fundus photograph

**Figure 2:**
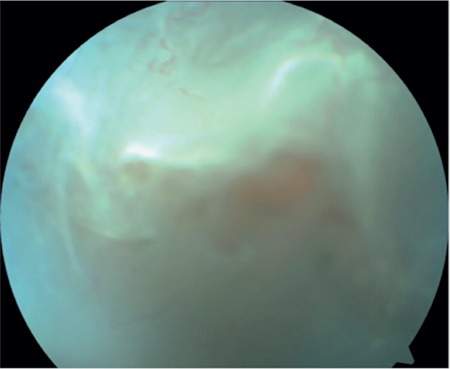
Preoperative appearance of the left retina in fundus photograph

**Figure 3:**
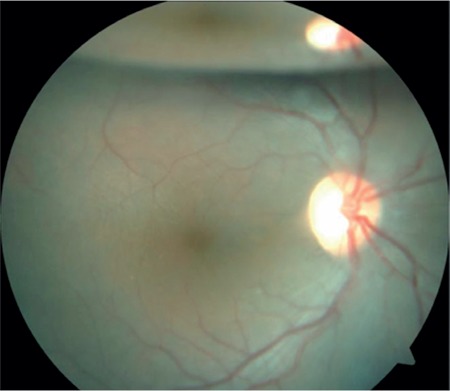
Right retinal appearance after scleral buckling in fundus photograph

**Figure 4:**
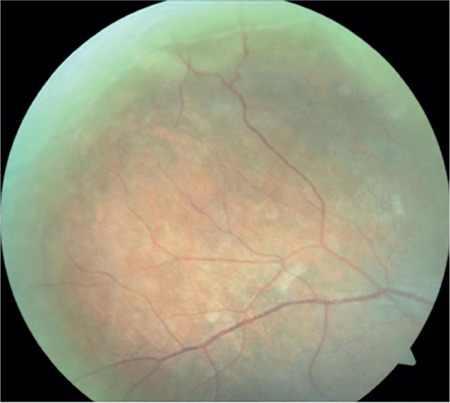
Right peripheral retinal appearance after scleral buckling in fundus photograph

**Figure 5:**
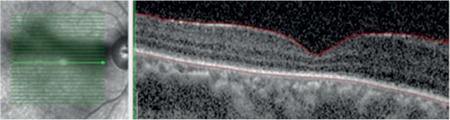
Optical cohorence tomography image of right eye after scleral buckling

**Figure 6:**
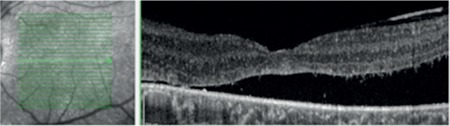
Optical cohorence tomography image of left eye after scleral buckling

**Figure 7:**
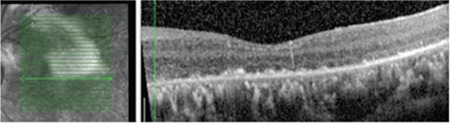
Optical cohorence tomography image of left eye after scleral buckling, pars plana vitrectomy, and silicone injection

A 17-year-old female patient presented to our clinic with complaints of low vision in both eyes for approximately 1 year. On ophthalmologic examination, her best corrected visual acuity (BCVA) was 0.1 in the right eye and 0.05 in the left eye. Bilateral subcapsular cataract was detected in anterior segment examination. Fundus examination revealed bilateral rhegmatogenous retinal detachment (Figures 1 and 2), membranous vitreous in the periphery, and chronic detachment with multiple holes and tears. There was proliferative vitreoretinopathy (PVR) grade C2 in the left eye. The patient exhibited the craniofacial structural features of Stickler syndrome (she did not consent to en face photography). Scleral buckling was performed in both eyes as an initial intervention and resulted in postoperative retinal attachment in the right eye (Figures 3, 4, 5). However, the procedure was insufficient for the left eye having PVR C2 (Figure 6). Phacoemulsification, intraocular lens implantation, and pars plana vitrectomy with silicone injection were performed in the left eye. Postoperatively the retina was reattached (Figure 7). Silicone extraction was done 6 months after pars plana vitrectomy. All procedures were performed by the same surgeon (I.A.).

**Figure 8:**
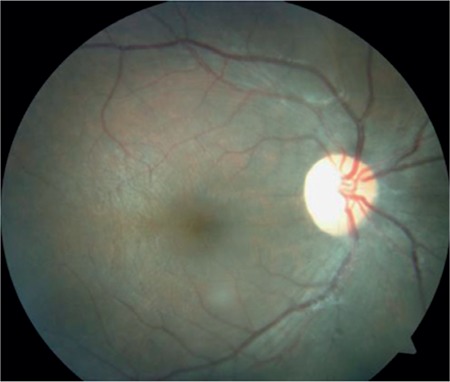
Fundus photograph showing right retinal appearance 3.5 years after scleral buckling

**Figure 9:**
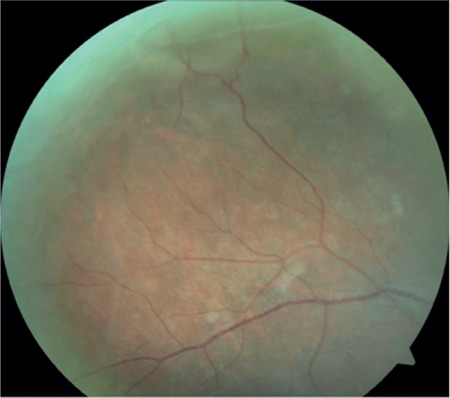
Fundus photograph showing appearance of the peripheral retinal of the right eye 3.5 years after scleral buckling

**Figure 10:**
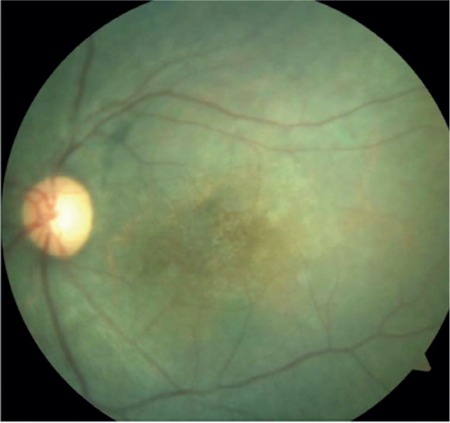
Fundus photograph showing left retinal appearance at 36 months after silicone extraction

**Figure 11:**
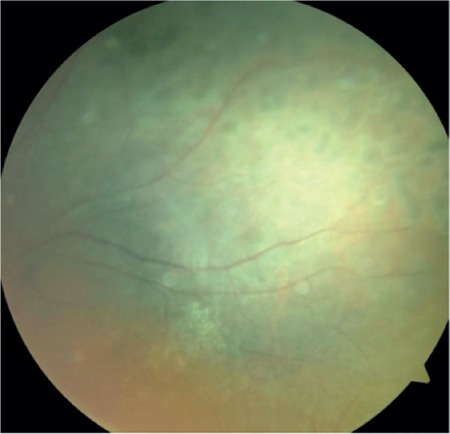
Fundus photograph showing appearance of peripheral retina of the left eye at 36 months after silicone extraction

**Figure 12:**
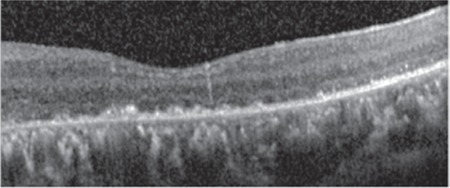
Optical cohorence tomography image of left eye 36 months after silicone extraction

In postoperative follow-up at about 3.5 years, the right eye had a BCVA of 0.6 and the retina was further attached (Figures 8, 9). In the left eye, the retina was attached and BCVA was 0.2 at 36-month follow-up after silicone extraction (Figures 10, 11, 12).

## Discussion

Stickler syndrome is a disease spectrum that has been investigated since Stickler et al.^6^ first described it in 1965. Though rare, it can involve serious complications. Ophthalmologic complications of the disease include high myopia, open-angle glaucoma due to dysgenesis at the drainage angle of the anterior chamber, cortical and subcapsular cataract, perivascular retinal lattice degeneration, and adhesive vitreous bands. These vitreoretinal changes cause giant retinal tears and subsequent rhegmatogenous retinal detachment.^7,8^ Although it is a rare condition, Stickler syndrome is the most common hereditary cause of rhegmatogenous retinal detachment.^9^ In a study from Turkey by Yararcan et al.^10^ including 6 individuals with Stickler syndrome in a single family, all of the patients had myopia of 10 diopters or greater, early onset cataract, chorioretinal atrophy, and vitreous liquefaction. Three of the 6 patients in that study had total retinal detachment, one had glaucoma, and one had phthisis bulbi.^10^ Abeysiri et al.^11^ presented the 17-year follow-up results of 30 eyes of 23 patients. In their study, the overall re-attachment rate was 78.4%, with rates of 67% for scleral buckling and 82.4% for pars plana vitrectomy. BCVA improved by 0.33 logarithm of minimum angle of resolution (logMAR) in patients that underwent scleral buckling and 0.32 logMAR in those that underwent vitrectomy.^11^In a study by Reddy et al.^12 ^including 16 eyes of 13 patients, the average age of patients with retinal detachment was 10.4 years and patients were followed for 94 months. Scleral buckling was performed in 5 patients (31%), pars plana vitrectomy in 7 patients (44%) and combined surgery in 4 patients (25%). The patients underwent a mean of 3.1 surgical interventions. In long-term follow-up, the retinal re-attachment rate was 100%, but 12 eyes (75%) developed PPV. The patient discussed in the present case had bilateral subcapsular cataract. Her fundus findings were consistent with those described in Stickler syndrome: fibrillary vitreous, lattice degenerations and adhesive vitreous bands, multiple retinal tears and holes, and PPV. The long-term outcomes of treatment were retinal re-attachment with BCVA of 0.6 in the right eye and 0.2 in the left eye. Scleral buckling and pars plana vitrectomy may be an effective and reliable treatment option for severe chronic retinal detachment due to Stickler syndrome.
